# A Grave Scandal

**Published:** 1919-04-26

**Authors:** 


### THE REGISTRATION OF NURSES.

### A Grave Scandal.

**[The Hydra was a serpent which inhabited a swamp in the country of the gods. It had no fewer than nine heads, and was finally overcome by Hercules, who discovered that for every head which he struck off two fresh ones sprang up in its place.—*The Classics.*]**

The Nurses' Registration Bill now before Parliament was, according to the preamble, prepared at the instance of the Central Committee which purports to represent nine Societies or Associations, "comprising not less than 30,000 medical practitioners and nurses." The President of the Local Government Board, on behalf of the Government, would appear to have adopted the entire family without making any inquiry into its parentage, or into the mental, moral, or spiritual health of any of its relations. Speaking for myself, I am sceptical of the potential benefits to be derived from any Registration Act which fails to confer a monopoly of employment. But there is no room for doubt as to the damage that may be wrought by placing an unsound Act on the Statute Book, and investing with authority persons and societies who never at any time represented the nursing profession. The nine Societies referred to are for the most part bogus, and the statement that they represent 30,000 members is fiction.

### What the Government has Failed to Realise.

Now, had this Bill been prepared at the instance of the State, the initial step would have been an impartial inquiry. A Commission would have been appointed and empowered to elicit certain very important facts which the authors of the Bill have been at pains to conceal, and the whole would have been published in a Blue Book for the information of all whom it might concern. It is the elementary right of every citizen to be fully informed on any subject of which the State is the parent, and therefore before this Bill becomes law it is imperative that the hydra-headed confederation known as the Central Committee should establish its claim to a membership of 30,000, or, in the alternative, forfeit the inflated representation which it claims on the Registration Council. Obviously such a suggestion as this ought to be responded to with enthusiasm and without delay by the Central Committee. To be able to demonstrate such a membership would be a triumphant answer to every critic, and would establish a claim for supremacy throughout the Empire. Moreover, if the facts are as alleged, the task is easy. Revenue is the true final test of membership. A chartered accountant, for a modest fee, would be able to issue a certificate showing how many separate members had paid subscriptions to each of the Societies enumerated in the memorandum during the year 1918. Of course, I except the British Medical Association, which does not purport to represent nurses, and with which I am not concerned.

Does anybody for a moment suppose that the Central Committee will honestly endeavour to establish its claim? I think not. The 30,000 members referred to are, I venture to suggest, potential members whose names are not yet enrolled, and whose subscriptions have not yet been paid! They are not the kind that a chartered accountant would be likely to recognise, and therefore the addition of a cipher or two is a matter of small consequence.

### The Two Irish Societies of the Central Committee.

May I take for illustration two of the Societies named—the Irish Nurses' Association and the Irish Nursing Board? These two are, in actual fact, one and the same. They resemble a clock having two dials—the same springs, wheels, and pendulum, but provided with an "Association" dial and a "Board" dial. The membership of both is practically identical, and consists of a handful of people resident in Dublin, with a few personal friends scattered up and down the country. It would be approximately accurate to say that neither of the Associations has any membership outside the Executive. I say approximately accurate because there are some involuntary subscribers. Certain prominent members of the Executive or Executives are also governors of a well-known Dublin hospital, every nurse in which finds it desirable to join, and whose subscription is deducted from her salary. So far, therefore, as being institutions representative of Irish nurses, both the I.N.A. and the I.N.B. are bogus, absolutely and utterly.

### A Monstrous Position.

To this pretence none could object so long as the Bill remained a private Bill. But where the State stands as sponsor the conditions automatically change. It is, I protest, monstrous that two bogus Societies, without inquiry or investigation, should be able to place two representatives on a State Registration Council for the United Kingdom, and their right to do so should be vigorously challenged, and without delay. Everybody in Ireland familiar with nursing affairs knows, in the first place, that when the College of Nursing opened its doors in Ireland the Irish Nursing Association consisted of a body of Dublin matrons, each of whom had a small following within her personal sphere of influence. But the Society derived no appreciable revenue from subscriptions. In the College of Nursing they saw a possible rival, and so they induced the Royal College of Surgeons, Ireland, to take them under its wing and form, with the addition of a few of its male members, the Irish Nursing Board. If this story is untrue in the letter or in the spirit the facts are easily ascertainable. Miss Carson Rae, St. Stephen's Green, Dublin, can supply

(*Continued on p.* 87.)

### The Registration of Nurses (*continued from p.* 88).

to any newspaper or member of Parliament a list of the names of the Executive of both Societies. She can also show how and by whom the Executive in each case is appointed, and she can prepare a list of members who paid subscriptions to either or to both during the year 1918. I notice that Sir Kingsley Wood, on behalf of Dr. Addison, said in committee that he was sure that neither the President nor any other member of the Committee desired to raise any question about "this or that Association"—"bodies which had done such valuable work." With great respect I venture to ejaculate—"Rubbish!"

### Away with Humbug: Parliament must have the Truth.

Sir Kingsley Wood is evidently an innocent and unsophisticated man. The Central Committee are past-masters of intrigue and artifice, and if I may venture to offer a suggestion it is that the Government should appoint a small impartial Committee of Inquiry to investigate the revenue-paying membership of this or that Association. Further, to obtain proof of the number of individual trained nurses who constitute their members, and of those who belong to more than one of the Societies or Associations the Central Committee claims to represent. Let us have the truth and the whole truth, and let there be no exceptions. British nurses deserve at least this measure of consideration. It is bad enough that they should be unrepresented. Let them not be misrepresented. Sir Kingsley Wood is pleased to be complimentary as to those Associations which have done "such valuable work." For one, I am unaware of what all this valuable work consists. It needs definition. Has any one of them increased the pay or diminished the hours of toil or raised the status of the working nurse? Has any one of them ever tried to do so? This Bill, if it becomes law, is not going to accomplish any of these things. But if the alleged Societies which it expresses and voices are to become the custodians of the British nurse's destiny, I venture to prophesy that the last state of that poor woman's fortunes will be worse than the first. And I cannot conceive of anything more depressing.

IERNE.

